# Inhibition of fucosylation by 2-fluorofucose attenuated acetaminophen-induced liver injury *via* its anti-inflammation and anti-oxidative stress effects

**DOI:** 10.3389/fphar.2022.939317

**Published:** 2022-09-01

**Authors:** Zhaoguo Liu, Mengjue Tu, Jianan Shi, Hong Zhou, Guoliang Meng, Jianguo Gu, Yuqin Wang

**Affiliations:** ^1^ Department of Pharmacology, School of Pharmacy and Key Laboratory of Inflammation and Molecular Drug Target of Jiangsu Province, Nantong University, Nantong, China; ^2^ Division of Regulatory Glycobiology, Institute of Molecular Biomembrane and Glycobiology, Tohoku Medical and Pharmaceutical University, Sendai, Miyagi, Japan

**Keywords:** fucosylation, 2-fluorofucose, acetaminophen, liver injury, oxidative stress

## Abstract

Fucosylation is a common glycan terminal modification, which has been reported to be inhibited by 2-fluorofucose (2FF) both *in vivo* and *in vitro*. The present study aimed to investigate the effect of 2FF on acetaminophen (APAP)-induced acute liver injury, and further clarified the possible mechanisms. In the present study, inhibition of fucosylation by 2FF relieved APAP-induced acute liver injury *in vivo*. Pretreatment with 2FF remarkably suppressed APAP-induced oxidative stress and mitochondria damage. 2FF markedly enhanced the nuclear translocation of nuclear factor erythroid 2-related factor 2 (Nrf2) and simultaneously promoted the expression of downstream proteins including HO-1 and NQO1. Furthermore, pretreatment with 2FF significantly suppressed the expression of inflammation-associated proteins, such as COX2 and iNOS. The data from lectin blot assay revealed that the alteration of α1,6-fucosylation was involved in APAP-induced acute liver injury. The second part of this study further confirmed that the enhancements to antioxidant capacity of 2FF pretreatment and α1,6-fucose deficiency were related to Nrf2/keap1 and NF-κB signaling pathways in HepG2 cells. Taken together, the current study suggested that 2FF might have a potential therapeutic effect for APAP-induced acute liver injury.

## Introduction

Acetaminophen (APAP) is an effective analgesic and antipyretic drug, which has had global and wide use since its application (Lee, 2017). Although it is generally considered to be safe at therapeutic doses, over dosage of APAP may result in hepatotoxicity and even acute liver failure (ALF). In developed countries, APAP-induced hepatotoxicity is the most common reason for drug-induced ALF (Bunchorntavakul & Reddy, 2018; Stravitz & Lee, 2019). N-acetylcysteine (NAC) is the only therapeutic option for patients with APAP-induced hepatotoxicity, however, poor efficacy has been made due to the narrow therapeutic window and adverse effects. Hence, great efforts have been devoted to understanding the pathological mechanism of APAP-induced hepatotoxicity and developing new drugs that are superior to NAC.

Oxidative stress is generally considered to be critical for APAP-induced hepatotoxicity. N-acetyl-P-benzoquinone (NAPQI), the metabolite of APAP, is accumulated in the liver after APAP overdose. Once glutathione (GSH) is depleted by excessive NAPQI, cellular proteins, especially mitochondrial proteins, covalent bind to sulfhydryl groups and result in severe mitochondrial oxidative damage and dysfunction, finally hepatocytes necrosis (Bunchorntavakul and Reddy., 2018). Increasing evidence indicates that many anti-oxidative signaling pathways are activated in the process of APAP-induced oxidative stress, and the nuclear factor erythroid 2-related factor 2 (Nrf2) is considered to be a key factor for the activation of some anti-oxidative systems *in vivo* (Wu et al., 2019). The activation of Nrf2 is regulated by redox status changes of the kelch-like ECH associated protein 1 (keap1), which can be induced by NAPQI (Klaassen & Reisman, 2010). When exposed to oxidative stimuli, Nrf2 dissociates from the Nrf2-keap1 complex in the cytoplasm, then transports to the nucleus and adjusts the genes encoding a series of antioxidant enzymes (Klaassen and Reisman., 2010; Ross and Siegel, 2021). Recent research shows that inhibition of Nrf2-keap1 interaction by a metal-based drug promotes Nrf2 nuclear translocation and alleviates APAP-induced liver injury (Li et al., 2021).

Undoubtedly, an acute inflammatory response is also induced by APAP overdose. Damage-associated molecular patterns derived from necrotic hepatocytes transcriptional activate proinflammatory cytokines including interleukin-1β (IL-1β), IL-10, IL-6 and tumor necrosis factor-α (TNF-α), which lead to the activation and recruitment of neutrophils in the damaged area of liver, and further aggravate hepatocytes necrosis in the early stage of APAP-induced liver injury (Yan, Huo, Yin, & Hu, 2018). Nuclear factor-κB (NF-κB), a transcription factor, is known to be a key factor in regulating inflammatory gene expression (Morgan & Liu, 2011). In addition, oxidative stress induced by overdosed APAP has the potential to regulate the activity of NF-κB. NF-κB activation, which is characterized by p65 subunit translocation, accelerates the expression of pro-inflammatory factors, including TNF-α, cyclooxygenase-2 (COX-2) and iNOS (Morgan and Liu., 2011; Zhang et al., 2016). Therefore, inhibiting NF-κB activation may be a possible target for therapeutic intervention of hepatotoxicity induced by APAP. It has been reported that the augment of endogenous omega-3 polyunsaturated fatty acids reduced NF-κB-mediated inflammation response and attenuated APAP-induced liver injury (Feng et al., 2018).

Glycosylation is a ubiquitous post-translational modification of most eukaryotic proteins, which has essential roles in diverse processes (Gloster and Vocadlo, 2012). Fucosylation, catalyzed by fucosyltransferases (FUTs), is a common glycan terminal modification, and the fucosylated structures widely exist in a variety of glycoproteins. However, altered fucosylated structures frequently appear during a variety of pathological processes, such as inflammatory response, tumorigenesis and metastasis (Li, Hsu, Mountz, and Allen, 2018). For instance, deficiency of core fucosylation (α1,6-fucose) that catalyzed by α1,6-fucosyltransferase (FUT8), reduced the pancreatic cancer cell proliferation and migration (Liang et al., 2021). In addition, accumulating studies have shown that dysregulation of fucosyltransferase 2 (FUT2) was associated with various human disorders, such as infection and chronic inflammatory diseases (Goto, Uematsu, and Kiyono, 2016).

Due to the effects on physiological and pathological processes, specific inhibitors of fucosylation may have important application value in basic research and treatment (Gloster and Vocadlo., 2012; Li et al., 2018). One such inhibitor, 2-fluorofucose (2FF), was recently reported to inhibit tumor cell adhesion, migration and proliferation by blocking fucosylation both *in vitro* and *in vivo* (Okeley et al., 2013; Carrascal et al., 2018). Pretreatment with 2FF inhibited the activation of NF-*k*B and expression of vascular cell adhesion molecule-1 in the livers of sickle cell disease (SCD) mice (Belcher et al., 2015). Additionally, our previous studies suggested that loss of core fucosylation inhibited chemical-induced hepatocellular carcinoma and liver regeneration (Wang et al., 2015; Wang et al., 2015), but the effects of 2FF and core fucosylation on APAP-induced acute liver injury were still unknown. Herein we estimated the effect of 2FF on APAP-induced acute liver injury *in vivo* and *in vitro*, and further expounded the possible mechanisms. All the results might provide the evidence to facilitate the development of an effective therapeutic strategy for APAP-induced acute liver injury.

## Materials and methods

### Animal treatment

The 6-week-old male ICR mice were randomly divided into five groups. (1) Control group: the mice were injected with saline intraperitoneally, and then received an intragastric administration of an equal volume of saline; (2) Model group: the mice were injected with saline intraperitoneally for 7 days, then received an intragastric administration of 400 mg/kg APAP (Ramachandran & Jaeschke, 2018); (3) 2FF control group: the mice were given an intraperitoneal injection of 150 mg/kg 2FF for 7 consecutive days, then received an intragastric administration of an equal volume of saline; (4) 2FF + APAP group: the mice were given an intraperitoneal injection of 150 mg/kg 2FF for 7 days (Belcher et al., 2015), and then received an intragastric administration of 400 mg/kg APAP; (5) APAP + NAC group, 1 h after APAP administration, the mice were given an intragastric administration of 500 mg/kg NAC. Four hours after APAP administration, the mice were sacrificed. The liver tissues and serum from different groups were taken for subsequent experiments. All detailed protocols were approved by the animal care and use committee of Nantong University.

### Cell treatment

The HepG2 cells were obtained from ATCC, USA, and the HepG2 FUT8 knockout (KO) cells were established as described (Y. Wang et al., 2015). All the cells were maintained in an incubator humidifier containing 5% CO2 at 37°C and were cultivated in DMEM medium which was supplemented with 10% fetal calf serum, streptomycin (100 g/ml) and penicillin (100 U/ml). The HepG2 wildtype (WT) cells were incubated with or without 100 μM 2FF for 48 h, meanwhile, the HepG2 FUT8 KO cells were not specially treated. Subsequently, the cells in APAP-treated groups were exposed to 20 mM APAP dilutions for 24 h.

### Reagents

2FF was provided by SynChem. Inc. (IL, United States). NAC was purchased from Sangon Biotech (Shanghai) Co., LTD. (Shanghai, China). Acetaminophen was provided by Abcam (CA, United States). The aspartate aminotransferase (AST), alanine aminotransferase (ALT), glutathione (GSH) and malondialdehyde (MDA) test kits were provided by Jiancheng Bioengineering Institute (Nanjing, China). The lactate dehydrogenase (LDH) kit, MTT cell proliferation and cytotoxicity kit and SOD activity kit were provided by Beyotime Biotechnology (Nantong, China). Reactive Oxygen Species (ROS) Fluorescent Probe-Dihydroethidium (DHE) was provided by Vigorousbio. CO, Ltd. (Beijing, China). The mouse TNF-α and IL-6 ELISA kits were obtained from ABclonal Technology Co., Ltd. (Wuhan, China). Aleuria aurantia lectin (AAL), wheat germ agglutinin (WGA) and concanavalin A (ConA) were provided by Vectorlabs, Inc. (CA, United States). Antibodies against Nrf2, NQO1, keap1, NF-κB p65 and iNOS were from Abcam (Cambridge, United Kingdom). Antibodies against glyceraldehyde-3-phosphate dehydrogenase (GAPDH), COX-2, HO-1 and Histone were from Proteintech (Wuhan, China).

### Hematoxylin & eosin staining

The liver tissues were harvested and washed with PBS on ice, then fixed in 4% paraformaldehyde. After dehydration, the specimens were embedded in paraffin and sectioned for H&E staining.

### Electron microscopy

For ultrastructural analysis of mitochondria, liver tissues were fixed with 2.5% glutaraldehyde and stored at 4°C. The specimens were post fixed with osmium in 0.2 M imidazole buffer, dehydrated, and then embedded in resin. Ultrathin cross sections were prepared by Lecia EM UC7, collected on formvar coated copper grids, and examined in a Hitachi HT7800 transmission electron microscope operated at 80 kV.

### Fluorescence probe-dihydroethidium staining

The ROS production was detected by DHE staining. Briefly, liver tissues were prepared for the frozen section. DHE was diluted with HEPES buffer, and the final concentration was 5 μM. After washing with 0.01 M PBS, the sections were incubated with diluted DHE at 37°C for 30 min. For HepG2 cells, 10^5^ cells in a 24-well plate were incubated with 5 μM DHE, and the nuclei were stained with DAPI. All the photos were taken with a laser confocal fluorescence microscope.

### MDA and GSH Assay

The liver and cells were homogenized on ice, and then supernatants were collected for the detections of MDA and GSH levels. The level of MDA was detected by thibabituric acid method, and the level of GSH was detected by dithiodinitrobenzoic acid method. The experiments were performed by following per under the manufacturer’s instructions.

### ELISA assay

The levels of IL-6 and TNF-α in mouse serum were detected by ELISA assay. The experiments were performed by respective kits according to the manufacturer’s protocol.

### Enzyme activity assay

According to the manufacturer’s protocol, the levels of ALT and AST were determined by Reitman-Frankel method with the respective kits, and the absorbance at 510 nm was measured. The activity of SOD was detected by WST-8 method with the corresponding commercial assay kit, then the absorbance at 450 nm was measured. Subsequently, the enzyme activities were calculated according to the formula. The total protein concentration was used as the standard for all the results in each sample.

### Western blot and lectin blot assay

Total protein was extracted from liver tissues and cells with RIPA lysis solution with phenylmethylsulfonyl fluoride (PMSF) on ice. All extracted proteins were quantified by BCA method and denatured at 95°C after mixing with loading buffer. The Western blot assay was carried out according to the standard protocol, as previously reported (Zeng, Hua, Wang, Zhang, and Xu, 2020). For lectin blot assay, the blocking buffer was replaced by 3% bovine serum albumin in TBST followed by specific lectins. Finally, an ABC Kit (Vectorlabs) was used for visualization of the bands. The quantification of band intensity was performed by Image-J analysis software.

### Statistical analysis

Data were presented as mean ± standard deviation. GraphPad Prism 7.0 software was utilized for data analysis. Comparison between multiple groups was performed by one-way ANOVA analysis of Tukey’s multiple comparison test, and *p* < 0.05 was considered statistically significant.

## Results

### Pretreatment with 2FF alleviated APAP-induced acute liver injury in mice

**FIGURE 1:**
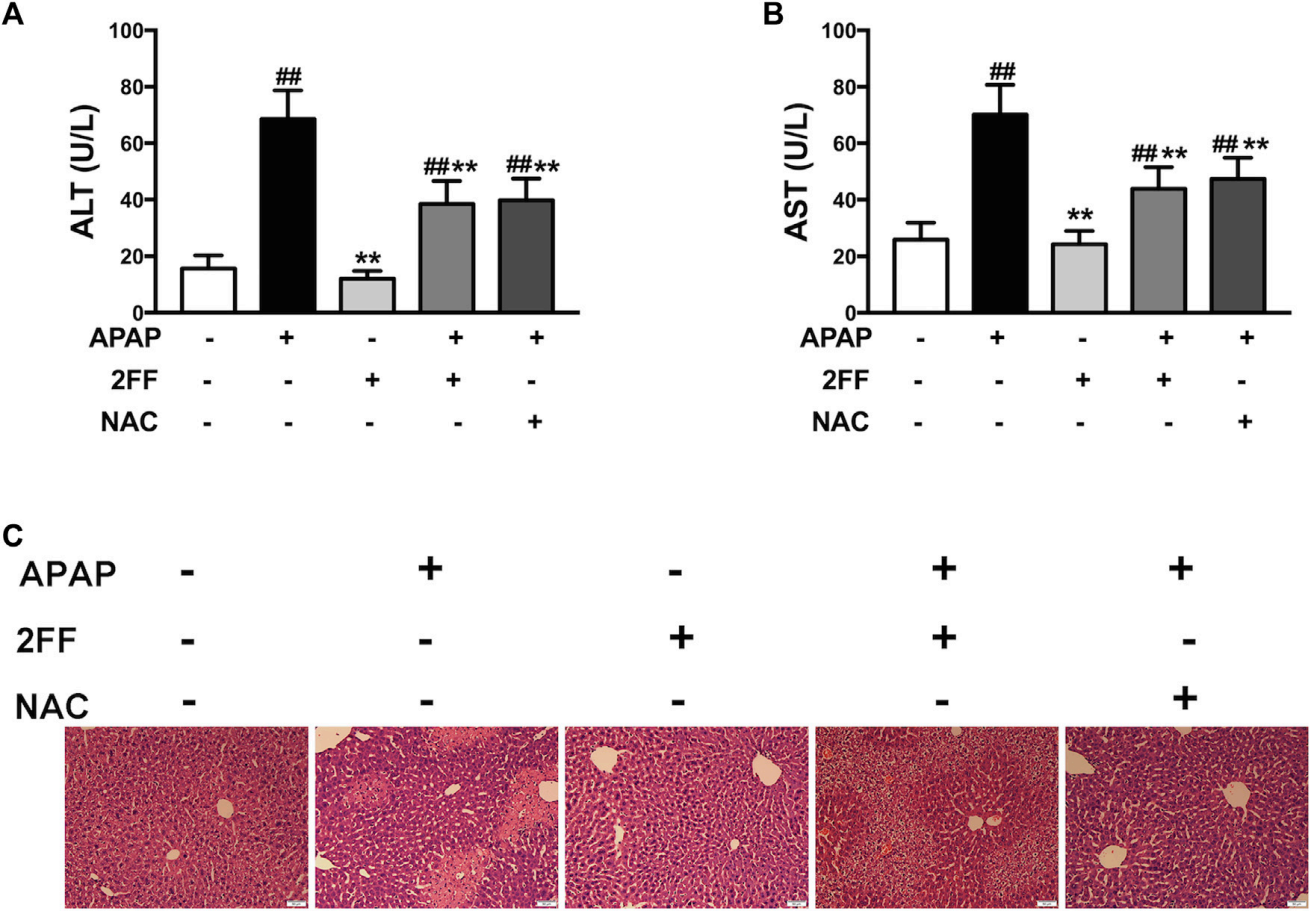
Pretreatment with 2FF alleviated APAP-induced acute liver injury in mice. The mice were treated with 150 mg/kg 2FF for 7 consecutive days, then an acute liver injury model was induced by an intragastric administration of 400 mg/kg APAP (0.1 ml/10 g, APAP group), and the control group was given equal amount of saline (0.1 ml/10 g). 4 h after APAP administration, the serum and the hepatic tissue was collected. Serum ALT **(A)** and AST **(B)** levels were measured for the evaluation of damage degree. **(C)**, H&E staining was used to observe the pathological injury of mouse liver (Bar = 50 µm). All results were expressed as mean ± SD, **p < 0.01 *vs*. the APAP-treated group; ^*##*^ *p* < 0.01 *vs*. the control group, *n* = 6.

Firstly, we evaluated the effect of 2FF on APAP-induced liver injury by detecting serum ALT and AST levels. In APAP model group, the levels of ALT and AST were remarkably increased compared to the control group, and treatment with 2FF or NAC significantly decreased ALT and AST levels (Figures 1A,B). As shown in Figure 1C, the hepatocytes were arranged orderly in the control group, however, were arranged disorderly and there was a large area of necrosis in the APAP model group. The damage was significantly relieved in 2FF and NAC treated groups. These results indicated that pretreatment with 2FF and NAC significantly alleviated APAP-induced acute liver injury in mice.

### Pretreatment with 2FF suppressed oxidative stress in APAP-treated mouse liver

**FIGURE 2:**
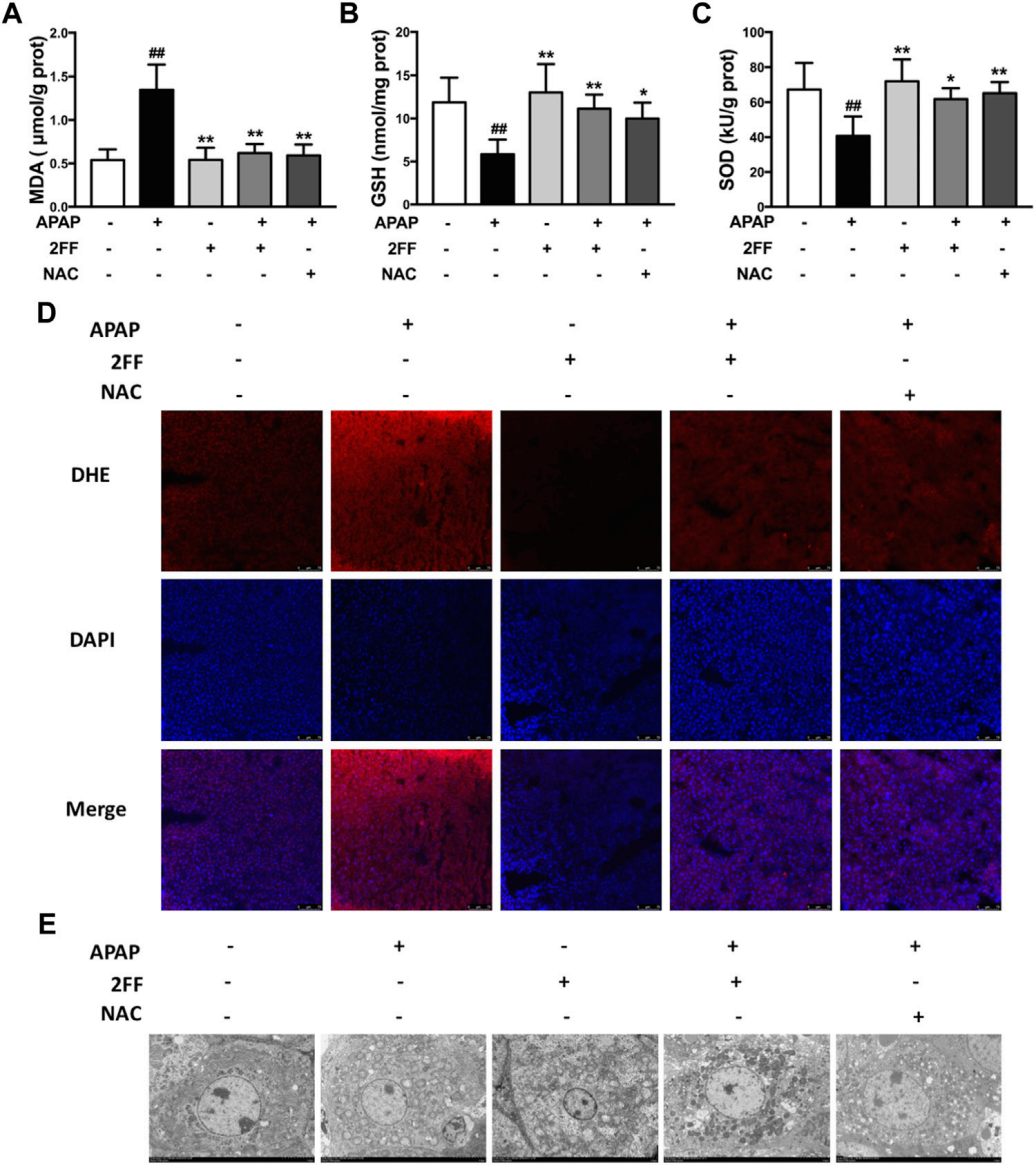
Pretreatment with 2FF suppressed oxidative stress in APAP-treated mouse liver. The mice were treated with 150 mg/kg 2FF for 7 consecutive days, then an acute liver injury model was induced by an intragastric administration of 400 mg/kg APAP (0.1 ml/10 g, APAP group), and the control group was given equal amount of saline (0.1 ml/10 g). The level of hepatic oxidative stress was evaluated by the measurements of MDA **(A)**, GSH **(B)** and the activity of SOD **(C)**. **(D)** The levels of ROS in liver were observed by DHE fluorescent dye staining, and the photos were taken by fluorescence microscope (Bar = 75 µm). **(E)**, The transmission electron microscope was used to observe the mitochondria (Bar = 5 µm). All results were expressed as mean ± SD, *p < 0.05, **p < 0.01 *vs*. the APAP-treated group; ^*##*^ *p* < 0.01 *vs*. the control group, *n* = 6.

Excessive ROS production has been considered as a key factor for APAP-induced hepatotoxicity (Bunchorntavakul and Reddy., 2018). In the APAP model group, the MDA level was significantly increased, and pre-treatment with 2FF and NAC remarkably suppressed the increase of MDA level induced by APAP (Figure 2A). In addition, the GSH levels and SOD activity were significantly decreased after APAP administration, while pre-treatment with 2FF and NAC evidently increased the GSH level as well as the SOD activity (Figures 2B,C). The ROS level in liver tissues was detected by DHE staining. Consistent with the above results, the fluorescence intensity in the APAP model group significantly increased, while 2FF and NAC dramatically reduced the fluorescence intensity (Figure 2D). To further confirm the effect of 2FF on the liver, transmission electron microscope was used to observe the mitochondria. As shown in Figure 2E, the structure of mitochondrial cristae was intact in the control group. Compared to the control group, APAP injection caused swelled and ruptured as well as the broken of the cristae in mitochondria. However, pre-treatment with 2FF and NAC notably improved the above changes in mitochondria. Collectively, these results suggested that inhibition of fucosylation by 2FF alleviated APAP-induced liver oxidative stress in mice.

### Pretreatment with 2FF affected Nrf2-mediated signal pathway in APAP-treated mice

**FIGURE 3:**
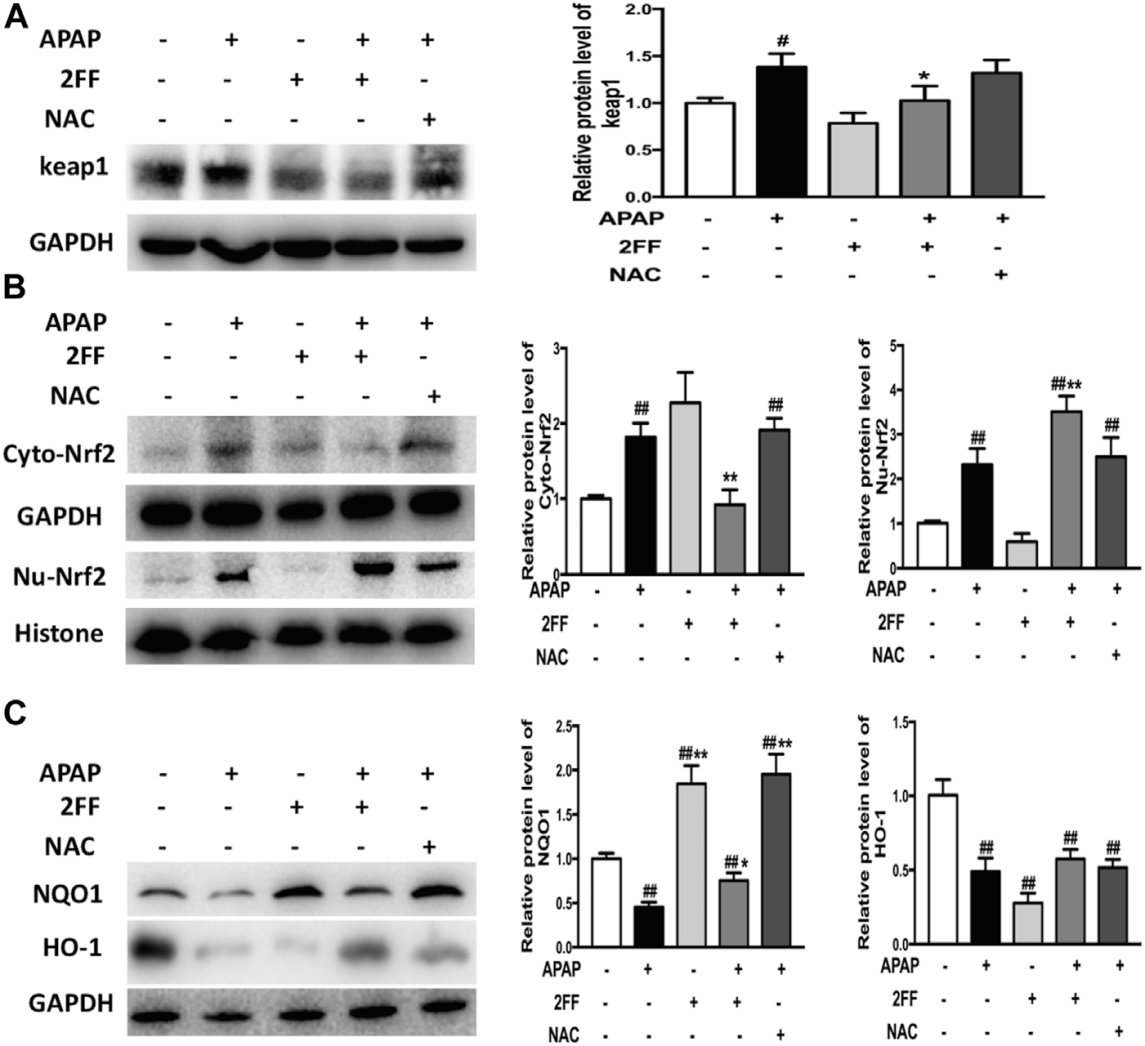
Pretreatment with 2FF affected Nrf2-mediated signal pathway in APAP-treated mice. The mice were treated with 150 mg/kg 2FF for 7 consecutive days, then an acute liver injury model was induced by an intragastric administration of 400 mg/kg APAP (0.1 ml/10 g, APAP group), and the control group was given equal amount of saline (0.1 ml/10 g). Western blotting analysis was used to detect the protein levels of keap1**(A)**, Cyto-Nrf2, Nu-Nrf2 **(B)**, NQO1 and HO-1**(C)** in mice liver. GAPDH or Histone was used as an internal control. The relative protein expression was quantified and statistically analyzed by densitometric analysis. All results were expressed as mean ± SD, *p < 0.05, **p < 0.01 *vs*. the APAP-treated group; ^*#*^ *p* < 0.05, ^*##*^ *p* < 0.01 *vs*. the control group, *n* = 6.

Nrf2/keap1 system is an important antioxidant system *in vivo* and is also plays crucial roles in APAP-induced hepatotoxicity (Klaassen and Reisman., 2010; Zhang et al., 2016). To elucidate the possible mechanism of 2FF against APAP-induced oxidative stress in the liver, the protein levels of keap1, Nrf2, NQO1 and HO-1 were determined by Western blot assay. Pretreatment with 2FF inhibited the protein expression of keap1 in APAP-induced mice liver (Figure 3A). APAP administration remarkably increased the expression of Nrf2 in cytoplasm and in nucleus, however, pre-treatment with 2FF significantly increased the level of Nrf2 in the nucleus and decreased the level of Nrf2 in the cytoplasm (Figure 3B). Moreover, as shown in Figure 3C, the protein levels of NQO1 and HO-1 were significantly decreased after APAP treatment, and pre-treatment with 2FF dramatically increased the protein expression NQO1. Unexpected, the decreased HO-1 expression induced by APAP was slightly enhanced by 2FF pre-treatment, but there was no significant statistical significance compared with the model group.

Altogether, the above data suggested that the protective effect of 2FF against APAP-induced hepatotoxicity, at least partly, by regulating the Nrf2/keap1 signalling pathway.

### Pretreatment with 2FF inhibited inflammatory response in APAP-induced acute liver injury

**FIGURE 4:**
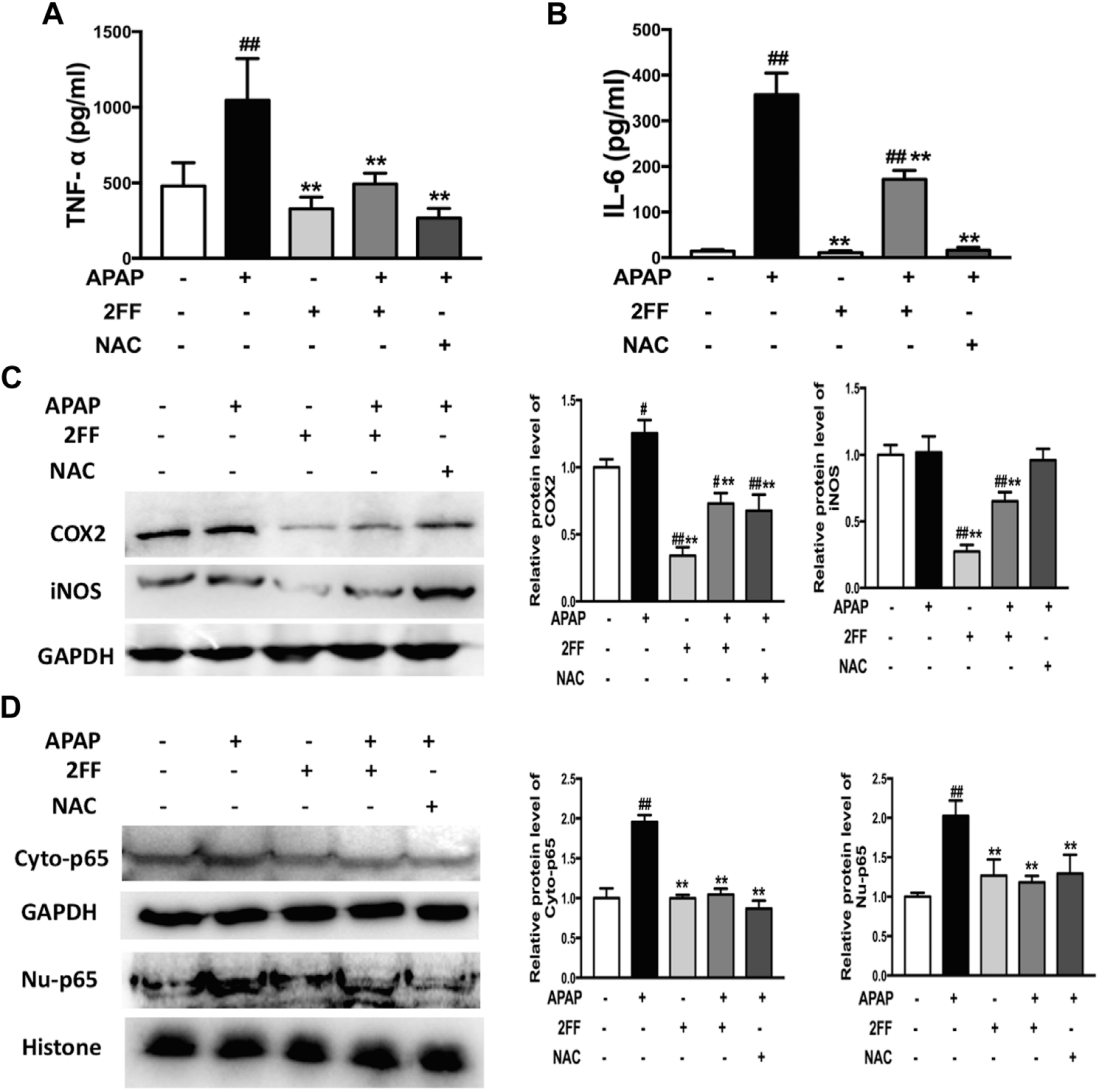
Pretreatment with 2FF inhibited inflammatory response in APAP-induced acute liver injury. The mice were treated with 150 mg/kg 2FF for 7 consecutive days, then an acute liver injury model was induced by an intragastric administration of 400 mg/kg APAP (0.1 ml/10 g, APAP group), and the control group was given equal amount of saline (0.1 ml/10 g). ELISA assay was used to determine the levels of serum TNF-⍺ **(A)** and IL-6 **(B)**. Western blotting analysis was used to detect the protein levels of COX2, iNOS**(C)**, Cyto-p65 and Nu-p65 **(D)** in mice liver. GAPDH or Histone was used as an internal control. The relative protein expression was quantified and statistically analyzed by densitometric analysis. All results were expressed as mean± SD, **p < 0.01 vs. the APAP-treated group; ^#^ *p* < 0.05, ^##^ *p* < 0.01 vs. the control group, *n* = 6.

Aseptic inflammation has been shown to play an important role in the early stage of APAP-induced hepatotoxicity (Yan et al., 2018). In this study, the serum levels of TNF-α and IL-6 significantly increased after APAP administration and were remarkably decreased by pre-treatment with 2FF and NAC (Figures 4A,B). In addition, COX-2 and iNOS protein expressions are usually induced in inflammatory environment. In Figure 4C, the protein expression of COX-2 in the APAP model group was significantly increased, and pre-treatment with 2FF and NAC notably inhibited the COX-2 expression. In addition, 2FF pre-treatment suppressed the protein expression of iNOS, even though no statistical difference was shown between control group and model group. Moreover, both in cytoplasm and nucleus, the protein levels of NF-κB p65 were induced by APAP, and 2FF remarkably decreased the protein levels of NF-κB p65 in cytoplasm and nucleus (Figure 4D). Collectively, these results indicated that 2FF alleviated APAP-induced aseptic inflammation in mouse liver, and the mechanism might be due to the regulation of NF-κB signalling pathway.

### Alterations of the *N*-linked glycans in APAP-induced acute liver injury

**FIGURE 5:**
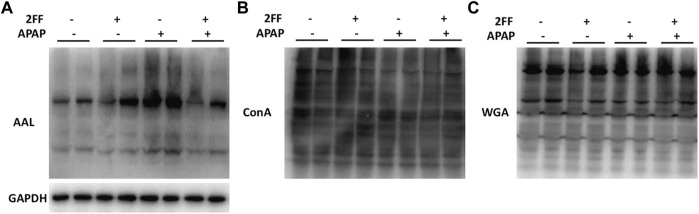
Alterations of the N-linked glycans in APAP-induced acute liver injury. The mice were treated with 150 mg/kg 2FF for 7 consecutive days, then an acute liver injury model was induced by an intragastric administration of 400 mg/kg APAP (0.1 ml/10 g, APAP group), and the control group was given equal amount of saline (0.1 ml/10 g). **(A)**, Lectin blot analysis using AAL was performed to recognize α1,6-fucose. **(B)**, Lectin blot analysis using ConA was performed to recognize α-linked mannose and glucose. **(C)**, Lectin blot analysis using WGA was performed to recognize N-Acetyl glucosamine. GAPDH was used as a loading control.

Aberrant glycosylation frequently occurs in various malignant tumors, nevertheless, the alterations of glycosylation in APAP-induced acute liver injury remain to be determined. Therefore, the levels of glycosylation in liver tissue were further detected by lectin blot assay in this study. The aleuria aurantia lectin (AAL), which specifically recognizes core fucose residue of branched *N*-glycans, was dramatically induced after APAP administration, and the enhancement was eliminated by 2FF (Figure 5A). Moreover, APAP administration showed no obvious effect on the levels of α-linked mannose and glucose and N-Acetyl glucosamine (GlcNAc) in liver tissue, which were specifically recognized by ConA and WGA, respectively (Figures 5B,C). Therefore, the above observations suggested that the alteration of α1,6-fucosylation was involved in APAP-induced acute liver injury.

### 2FF and FUT8 deficiency relieved APAP-induced cell damage in HepG2 cells

**FIGURE 6:**
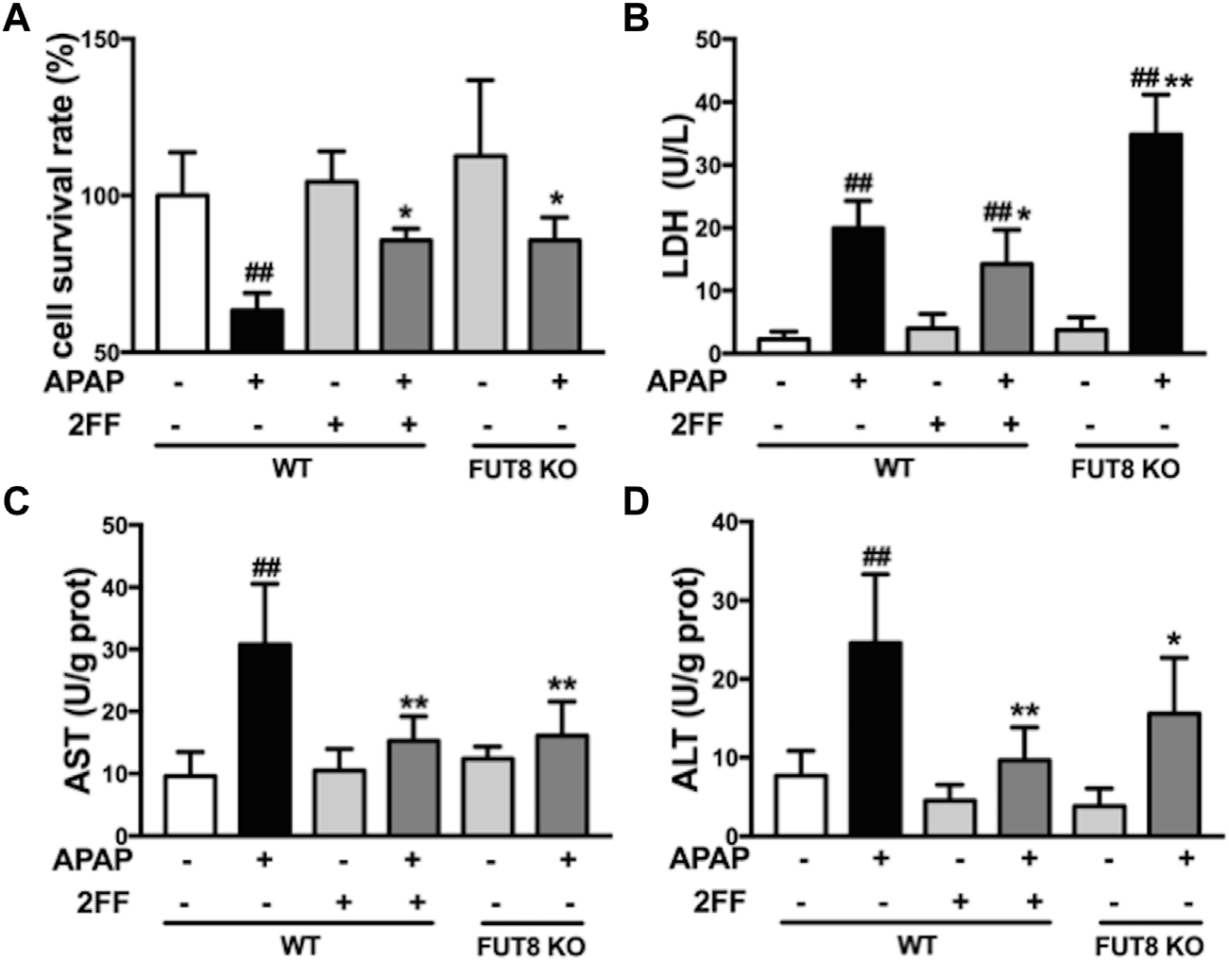
2FF and FUT8 deficiency relieved APAP-induced cell damage in HepG2 cells. After incubating with or without 100 μM 2FF for 48 h, the HepG2 WT and HepG2 FUT8 KO cells were treated or not with 20 mM APAP dilutions for 24 h **(A)**, MTT assay was used to evaluate cell viability. The levels of LDH **(B)**, ALT **(C)** and AST **(D)** were detected for evaluating the damage of cells. Data were expressed as mean ± SD, ^*##*^ *p* < 0.01, *vs*. HepG2 WT control group; *p < 0.05, **p < 0.01, *vs*. APAP-treated HepG2 WT group, *n* = 3.

The abovementioned results showed that the increase of α1,6-fucosylation might be related with the process of APAP-induced acute liver injury in mice. Since FUT8 is the only fucosyltransferase that catalyzes the formation of core fucosylation in mammals, therefore, the human hepatoma HepG2 wild-type (WT) and HepG2 FUT8 KO cells were used to clarify the hepatic protective mechanism of 2FF *in vitro*. MTT assay showed that the survival rate of HepG2 WT cells significantly decreased when exposed to APAP, while 2FF pretreatment significantly improved the survival rate of HepG2 WT cells, and the same results were obtained in FUT8 KO cells (Figure 6A). Similarly, 2FF markedly decreased the level of LDH in APAP-treated HepG2 WT cells. Unexpected, the LDH level in the HepG2 FUT8 KO group was significantly enhanced by APAP (Figure 6B). Moreover, the supernatant levels of AST and ALT in APAP-treated WT cells were significantly increased, while 2FF pretreatment and FUT8 gene deficiency significantly relieved the increase induced by APAP (Figures 6C,D). These data suggested that inhibition of fucosylation, especially core fucosylation, effectively alleviated APAP-induced cell damage.

### 2FF and FUT8 deficiency relieved APAP-induced oxidative stress in HepG2 cells

**FIGURE 7:**
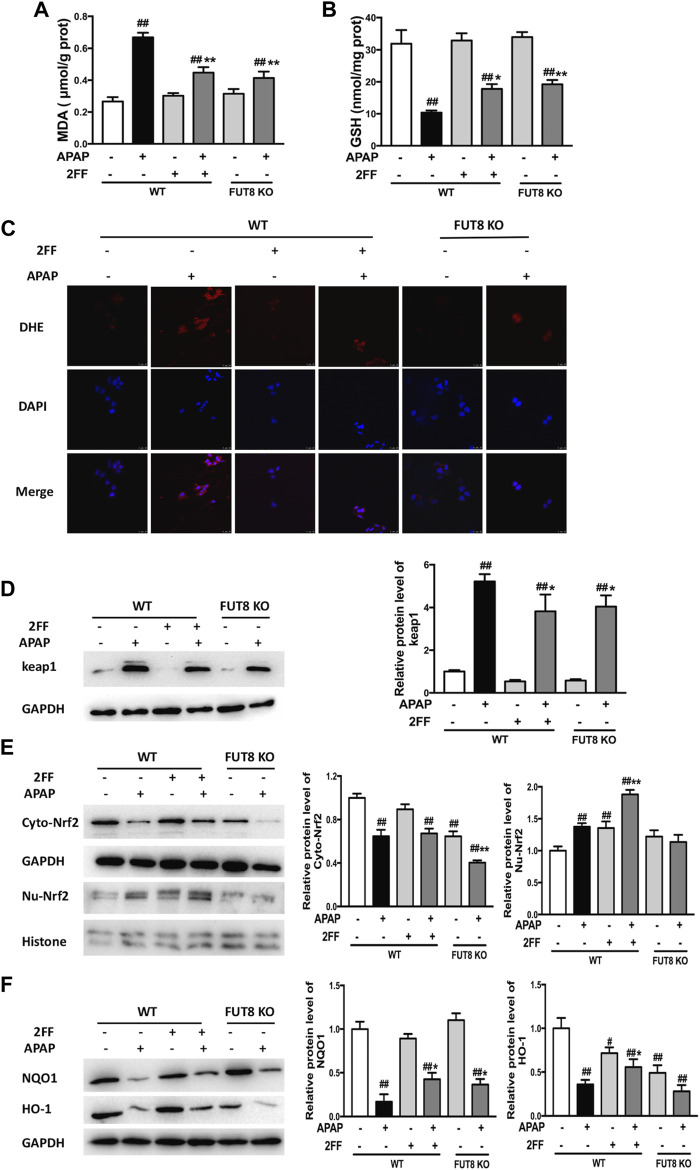
2FF and FUT8 deficiency relieved APAP-induced oxidative stress in HepG2 cells. After incubating with or without 100 μM 2FF for 48 h, the HepG2 WT and HepG2 FUT8 KO cells were treated or not with 20 mM APAP dilutions for 24 h. The levels of MDA **(A)** and GSH **(B)** were determined for evaluating the level of oxidative stress in cells. **(C)**, ROS in the cells was detected using a laser confocal microscope with DHE fluorescent dye (red). DAPI was used for nuclear staining (blue) (Bar = 25 μm). Western blotting analysis was used to detect the protein levels of keap1 **(D)**, cytoplasm Nrf2, nuclear Nrf2 **(E)**, NQO1 and HO-1 **(F)** in different group. GAPDH or Histone was used as an internal control. The relative protein expression was quantified and statistically analyzed by densitometric analysis. Data were expressed as the mean ± SD, ^*#*^ *p* < 0.05, ^*##*^ *p* < 0.01, *vs*. HepG2 WT control group; *p < 0.05, **p < 0.01, *vs*. APAP-treated HepG2 WT group, *n* = 3.

Compared to the WT cells without any treatment, the MDA level significantly increased in APAP model group, and notably suppressed by 2FF and FUT8 gene knockout (Figure 7A). Meanwhile, the GSH level in APAP-treated cells was much lower than that in control cells, and 2FF treatment and FUT8 deficiency evidently increased the level of GSH (Figure 7B). The ROS level in the cells was detected by DHE staining, and the result was consistence with the above results (Figure 7C). In order to elucidate the protective mechanism of 2FF on APAP-induced oxidative stress, Western blot assay was used to determine the protein levels of keap1, cytoplasm and nuclear Nrf2. Pretreatment with 2FF significantly reduced the elevation of keap1 protein expression induced by APAP, subsequently increasing the protein level of nuclear Nrf2 (Figures 7D,E). After APAP induction, there was no significant enhancement of nuclear Nrf2 level in HepG2 FUT8 KO cells, even APAP-induced increase of keap1 and cytoplasm Nrf2 was markedly suppressed by FUT8 gene deficiency in HepG2 cells (Figures 7D,E). Moreover, NQO1 and HO-1 protein levels remarkably decreased after APAP treatment both in HepG2 WT and HepG2 FUT8 KO cells. Unexpectedly, the protein levels of NQO1 were enhanced in 2FF-treated and FUT8 KO groups after APAP administration, while the change of HO-1 protein levels was not consistent with that of NQO1 (Figure 7F). Based on these results, it could be inferred that 2FF and loss of core fucose effectively reduced the oxidative stress induced by APAP, and the mechanism was partially achieved by affecting the Nrf2/keap1 signaling pathway.

### 2FF and FUT8 deficiency influenced APAP-induced inflammatory response in HepG2 cells

**FIGURE 8:**
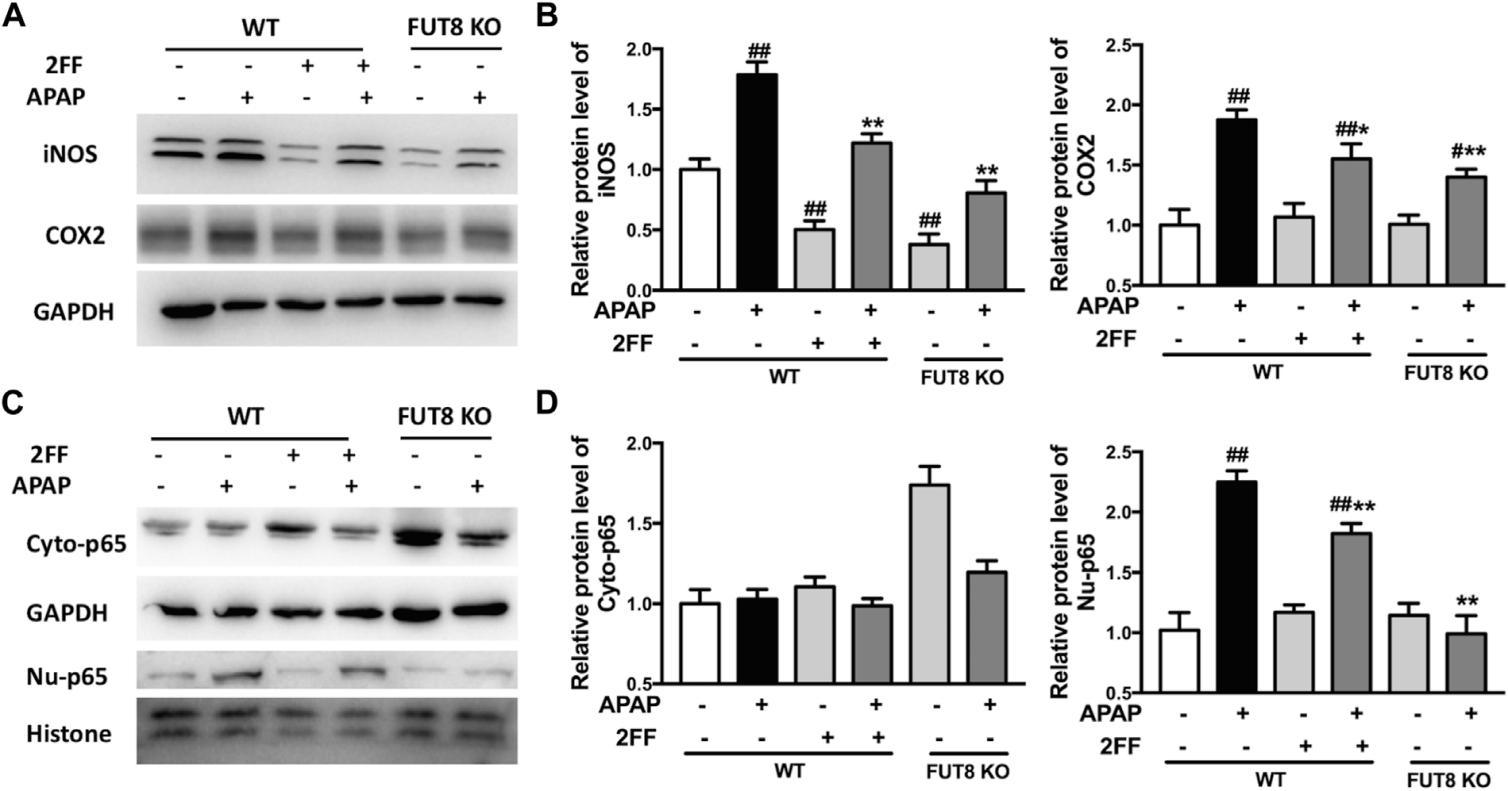
2FF and FUT8 deficiency influenced APAP-induced inflammatory response in HepG2 cells. After incubating with or without 100 μM 2FF for 48 h, the HepG2 WT and HepG2 FUT8 KO cells were treated or not with 20 mM APAP dilutions for 24 h. Western blotting analysis was used to detect the protein levels of iNOS, COX2 **(A,B)**, Cyto-p65 and Nu-p65 **(C,D)** in different group. GAPDH or Histone was used as an internal control. The relative protein expression was quantified and statistically analyzed by densitometric analysis. Data were expressed as the mean ± SD, ^*#*^ *p* < 0.05, ^*##*^ *p* < 0.01, *vs*. HepG2 WT control group; *p < 0.05, **p < 0.01, *vs*. APAP-treated HepG2 WT group, *n* = 3.

To confirm the effects of 2FF and core fucose on APAP-induced inflammatory response in HepG2 cells, Western blot assay was performed to detect the protein expression of iNOS and COX-2. The protein levels of iNOS and COX-2 were significantly up-regulated by APAP administration, and 2FF remarkably inhibited the expression of iNOS and COX-2 in HepG2 WT cells. Meanwhile, the enhancement of iNOS and COX-2 by APAP were markedly suppressed in FUT8 KO cells (Figures 8A,B). In addition, APAP remarkably up-regulated nuclear NF-κB p65 levels, and the increase was significantly inhibited by 2FF pretreatment and FUT8 deficiency (Figures 8C,D). These results suggested that 2FF and deletion of core fucose reduced APAP-induced inflammatory response might be related with the NF-κB signaling pathway.

## Discussion

APAP-induced hepatotoxicity is considered to be the main cause of ALF in western countries, which has attracted a great deal of clinical concern. Thus, understanding the underlying mechanism of APAP-induced acute liver injury is necessary to design its new therapeutic strategies. In present study, we evaluated the protective effects of 2FF in APAP-induced acute liver injury, and further expounded the potential mechanisms of core fucosylation during the pathological process for the first time.

Excessive ROS production and decreased antioxidant defense system are considered key characteristics of APAP-induced hepatotoxicity (Ramachandran & Jaeschke, 2018). In our study, APAP-induced ROS accumulation and GSH reduction were relieved by 2FF administration *in vivo* (Figure 2). Similar results were observed in HepG2 cells *in vitro* (Figure 7). Therefore, we speculated that suppression of APAP-induced oxidative stress might be critical for the protective effect of 2FF on APAP-injured liver. As one of the most important antioxidant systems, the activation of Nrf2/Keap1 system is associated with the alleviation of APAP-induced hepatotoxicity (Klaassen and Reisman., 2010). In current study, 2FF pretreatment significantly increased nuclear Nrf2 level in liver tissue of APAP-treated mice. Moreover, the downstream antioxidant targets such as Nrf2 and NQO1 protein expressions were remarkably induced by 2FF administration in APAP-treated mice, which effectively alleviated oxidative stress in liver. The data in HepG2 cells were consistent with the results *in vivo*. Unexpected, the protein expression of HO-1 was not enhanced by 2FF either *in vivo* or *in vitro*. Actually, Nrf2 is not the unique pattern to regulate the expression of HO-1 and NQO1 (Zhang et al., 2016). For example, convincing evidence exists for HO-1 regulation by some other transcription factors, including heat-shock factor, activator protein-1, and NF-κB families (Paine, Eiz-Vesper, Blasczyk, and Immenschuh, 2010). Thus, the levels of HO-1 and NQO1 are often inconsistent in different patterns.

In the early stage of APAP-induced liver injury, aseptic inflammation is another important pathological process, and is closely associated with oxidative damage and further aggravates liver damage (Ramachandran and Jaeschke., 2018). In APAP-treated mice, TNF-α and IL-6 levels were significantly decreased by 2FF pretreatment. Furthermore, the abnormal increase of COX-2 and iNOS protein expression in APAP-treated mice was also markedly suppressed by the 2FF treatment. These results suggested that inhibition of aseptic inflammatory response might be important for the protective effect of 2FF on the liver. NF-κB is considered to be a key regulator of inflammatory response, which is also involved in the process of oxidative stress (Morgan and Liu., 2011). Excessive ROS activates NF-κB-mediated pathways, meanwhile, NF-κB affects the expression of antioxidant proteins. In our study, APAP-induced enhancements of nuclear NF-κB p65 were remarkably suppressed after 2FF pretreatment. All the results proved that NF-κB-mediated pathway was one of the possible mechanisms for protective effect of 2FF on liver.

In current study, AAL level significantly increased in the liver of APAP-treated mice, suggesting that abnormal core fucosylation might be involved in the pathological process of APAP-induced hepatotoxicity. As expected, the HepG2 cells either pretreated with 2FF or loss of core fucose effectively attenuated APAP-induced oxidative stress. Subsequently, the protein expression of NQO1, HO-1, iNOS and COX2 in HepG2 cells were consistent with those *in vivo*. All the results showed that fucosylation, especially core fucosylation, played an important role in the pathological process of APAP-induced acute liver injury.

Previous studies on core fucosylation have shown that it is crucial for dimer formation, ligand-binding affinity and downstream signaling of glycoprotein receptors, such as epidermal growth factor receptor (EGFR) (Wang, Gu, Ihara, Miyoshi, Honke, and Taniguchi, 2006; Y. Wang et al., 2015). The absence of core fucosylation reduced the binding affinity of EGF and EGFR, and then suppressed EGFR-mediated downstream pathways (Wang et al., 2006; Liu et al., 2011). Recently, it has been reported that EGFR was activated by GSH depletion in the early phase of APAP-induced liver injury (B.Bhushan and Michalopoulos, 2020). Canertinib, an inhibitor of EGFR, has been reported to result in the early inhibition of APAP-induced EGFR activation and liver necrosis (Bhushan et al., 2017). Thus, we postulated that one possible mechanism of 2FF alleviating APAP-induced acute liver injury might be due to the inhibition of EGFR-mediated pathways after core fucose deletion.

Nevertheless, since the effects of fucosylation on biological functions mediated by a variety of proteins are complex, it is still possible that other mechanisms are involved in the effects of 2FF on acute liver injury induced by APAP. For instance, 2FF has been reported to have anti-inflammatory effect by inhibiting the activation of NF-kB in SCD mice (Belcher et al., 2015). In addition, the interferon (IFN)-β production induced by lipopolysaccharide is significantly suppressed in FUT8-deficient mouse embryonic fibroblasts, which is attributed to the impaired TLR4/CD14 internalization caused by core fucose deletion (Iijima et al., 2017). In the present study, the increased expression of iNOS, COX2 and nuclear NF-κB p65 induced by APAP were significantly suppressed by 2FF pretreatment and FUT8 deficiency (Figures 4, 8), which was consistent with the previous studies. The data from these studies provided the evidence that 2FF treatment and core fucose deficiency inhibited inflammation both *in vivo* and *in vitro*, however, we also found that effects of core fucosylation on the peripheric inflammation were different from the neuroinflammation in previous study (Lu et al., 2019). In microglia and astrocytes, deficiency of core fucose resulted in enhancements of pro-inflammatory signaling pathways and inhibitions of anti-inflammatory signaling pathways (Lu et al., 2019). As mentioned above, the role of 2FF and core fucosylation in APAP-induced aseptic inflammation is complicated, and the detailed mechanisms require further studies.

In conclusion, pharmacological inhibition of fucosylation attenuated APAP-induced acute liver injury, and its mechanism might be due to the inhibition of oxidative stress and aseptic inflammation. Although further studies are needed, our study supported the emerging notion that core fucosylation was pivotal in the early phase of APAP-induced liver injury.

## Data Availability

The original contributions presented in the study are included in the article/supplementary material, further inquiries can be directed to the corresponding authors.
